# Evaluating automated or artificial intelligence search tools for evidence synthesis

**DOI:** 10.5195/jmla.2026.2341

**Published:** 2026-04-01

**Authors:** Robin Featherstone

**Affiliations:** 1 robin.featherstone@cda-amc.ca, Acting Director, Research Information Services, Canada's Drug Agency, Ottawa, Ontario

**Keywords:** Information Sciences, Information Storage and Retrieval, Review Literature as Topic, Artificial Intelligence, Generative Artificial Intelligence, Large Language Models, Automation

## Abstract

To advance information retrieval science for producing evidence syntheses at Canada's Drug Agency, the Research Information Services team developed a replicable process to evaluate automated or artificial intelligence (AI) search tools. The team inventoried 51 tools in the fall of 2023 and built a flexible evaluation instrument to inform adoption decisions and enable comparison between tools. Building on this foundational evaluation work, the team further conducted a comparative analysis on three top-ranked tools in the fall of 2024. The investigation confirmed that these automated or AI tools have inconsistent and variable performance for the range of information retrieval tasks performed by Information Specialists at Canada's Drug Agency. Implementation recommendations from this study informed a “fit for purpose” approach where Information Specialists leverage automated or AI search tools for specific tasks or project types.

**Context, aims, and significance of the virtual project:** Canada’s Drug Agency is a pan-Canadian health organization that provides independent evidence and advice so health system leaders can make informed drug, health technology, and health system decisions. The Research Information Services (RIS) team at Canada’s Drug Agency consists of masters in library and information science (MLIS)-credentialled Information Specialists (ISs) who conduct systematic and abbreviated literature searches for evidence reports produced by the agency.

Given the volume of untested and rapidly evolving automated or Artificial Intelligence (AI) tools for information retrieval, RIS initiated a project to identify, evaluate, and recommend tools to incorporate into IS workflows. Canada’s Drug Agency funded this work, and the RIS project team consisted of one manager, six ISs, a statistician, and an IS methodologist.

Through this work, the RIS team developed a sustainable evaluation process to stay abreast of emerging search technologies and a customizable instrument to be used by other evidence synthesis producers. This evaluation work was foundational to the subsequent conduct of a comparative analysis of automated or AI search tools and the development of implementation recommendations for Canada’s Drug Agency and all evidence synthesis producers.

**Brief description of the virtual project:** For the primary project phase, RIS compiled an inventory of 51 search tools, assessed and ranked these tools, and built a flexible evaluation instrument to prioritize tools for further testing. RIS also conducted a comparative analysis of three top-rated tools from the team’s evaluations and published recommendations that showed the variable performance of AI search tools and identified cases where their use was warranted for information retrieval tasks.

**Technology used:** RIS’ inventory relied on curated collections of search tools: the Systematic Review Toolbox, and York Health Economics Consortium’s list of citation analysis tools. RIS supplemented the inventory with tools identified through a weekly research feed generated from a Semantic Scholar research library.

RIS built our inventory and our evaluation instrument using Microsoft Excel. The comparative analysis for this project tested Lens.org, a metadata aggregator of scholarly and patent literature; SpiderCite, a citation searching tool now available through the TERA suite of automation tools for systematic reviews; and Microsoft Copilot, a generative AI chatbot built upon OpenAI’s GPT-4 LLM5.

**Advantages, limitations, and impact:** An advantage to the evaluation instrument is that it allowed RIS to compare tools as they become available and to prioritize tools that the team determined to be the most likely to benefit their work. A limitation of this work is that the evaluation criteria did not account for potential sources of bias in the tools (e.g., language bias). The project team acknowledges the need for ISs to interrogate sources of biases and for tool developers to report these transparently. Despite this limitation, the flexible evaluation instrument may be modified to account for additional criteria by users – including potential sources of bias. With the development of evaluation approaches and implementation recommendations from testing, RIS’ work enables judicious use of innovative AI and automated technologies to enhance information retrieval for Canada’s Drug Agency and other evidence synthesis producers.

**Contact information:** requests@cda-amc.ca.

## LINKS

- Systematic Review Toolbox: https://www.systematicreviewtools.com/
- York Health Economics Consortium’s list of citation analysis tools: https://sites.google.com/york.ac.uk/yhectrainingpages/home/sept-2022-citation-analysis
- Semantic Scholar: https://www.semanticscholar.org/
- Artificial intelligence search tool evaluation instrument. Ottawa: Canada’s Drug Agency; 2024. https://www.cda-amc.ca/sites/default/files/AI-Search-Tool-Evaluation-Instrument_v1.1.xlsx Accessed 2025-08-28.
- Lens.org (“The Lens”): https://www.lens.org/
- SpiderCite: https://tera-tools.com/spidercite
- Microsoft Copilot: https://www.microsoft.com/en-ca/microsoft-copilot
